# Bioactive Poly(lactic acid)–Cocoa Bean Shell Composites for Biomaterial Formulation: Preparation and Preliminary In Vitro Characterization

**DOI:** 10.3390/polym13213707

**Published:** 2021-10-27

**Authors:** Andres J. Garcia-Brand, Maria A. Morales, Ana Sofia Hozman, Andres C. Ramirez, Luis J. Cruz, Alejandro Maranon, Carolina Muñoz-Camargo, Juan C. Cruz, Alicia Porras

**Affiliations:** 1 Department of Biomedical Engineering, School of Engineering, Universidad de los Andes, CR 1 No. 18A-12, Bogota 111711, Colombia; c.munoz2016@uniandes.edu.co (C.M.-C.); jc.cruz@uniandes.edu.co (J.C.C.); 2 Grupo de Diseño de Productos y Procesos (GDPP), Department of Chemical and Food Engineering, School of Engineering, Universidad de los Andes, CR 1 No. 18A-12, Bogota 111711, Colombia; ma.morales12@uniandes.edu.co (M.A.M.); as.hozman10@uniandes.edu.co (A.S.H.); ac.ramirez10@uniandes.edu.co (A.C.R.); lj.cruz@uniandes.edu.co (L.J.C.); 3 Structural Integrity Research Group, Department of Mechanical Engineering, School of Engineering, Universidad de los Andes, CR 1 No. 18A-12, Bogota 111711, Colombia; emaranon@uniandes.edu.co

**Keywords:** cocoa bean shell, poly(lactic acid), composites, natural fibers, biocompatibility, mechanical properties

## Abstract

The unique lignocellulosic and solvent-extractive chemical constituents of most natural fibers are rich in natural polymers and bioactive molecules that can be exploited for biomaterial formulation. However, although natural fibers’ main constituents have been already incorporated as material reinforcement and improve surface bioactivity of polymeric materials, the use of the whole natural fibers as bioactive fillers remains largely unexplored. Thus, we put forward the formulation of natural fiber filling and functionalization of biomaterials by studying the chemical composition of cocoa bean shells (CBS) and proposing the fabrication and characterization of polylactic acid (PLA) and CBS-based composite by solvent-casting. As was expected from previous studies of agro-industrial wastes, the main components of CBS were to cellulose (42.23 wt.%), lignin (22.68 wt.%), hemicellulose (14.73 wt.%), and solvent extractives (14.42 wt.%). Structural analysis (FTIR) confirms the absence of covalent bonding between materials. Thermal degradation profiles (DSC and TGA) showed similar mass losses and thermal-reaction profiles for lignocellulosic-fibers-based composites. The mechanical behavior of the PLA/CBS composite shows a stiffer material behavior than the pristine material. The cell viability of Vero cells in the presence of the composites was above 94%, and the hemolytic tendency was below 5%, while platelet aggregation increased up to 40%. Antioxidant activity was confirmed with comparable 2,2-diphe-277 nyl-1-picryl-hydrazyl-hydrate (DPPH) free-radical scavenging than Vitamin C even for PLA/CBS composite. Therefore, the present study elucidates the significant promise of CBS for bioactive functionalization in biomaterial-engineering, as the tested composite exhibited high biocompatibility and strong antioxidant activity and might induce angiogenic factors’ release. Moreover, we present an eco-friendly alternative to taking advantage of chocolate-industry by-products.

## 1. Introduction

Synthetic polymers (SP) have become a part of our daily routine; however, their extended use has brought several concerns about long-term effects, the sustainability of manufacturing technologies involved, and end-of-life disposal [1,2]. To address some of these issues, over the last decades, natural polymers (NP) from renewable resources have emerged as an attractive alternative due to their ease of degradation, low environmental impact, and high biocompatibility. This has resulted in a growing number of engineering applications, including packing, construction, and tissue-inspired biomaterials [2,3]. Besides these attractive attributes, these polymers exhibit superior chemical stability in different environments and comparable mechanical properties to those of commonly used SPs [3]. NP has shown excellent performance when used as a replacement of SP in many applications, including packaging (bags and foams) [4], agricultural mulch [5], and medical devices (i.e., scaffolds for regenerative medicine, synthesis of nano vehicles for drug delivery, etc.) [6]. In addition, some approaches have been successfully explored by using NPs as resorbable and biocompatible composite scaffolds for the treatment of damaged tissues, despite their absence of surface bioactivity [6,7].

The fabrication of natural polymeric materials to replace SP has increased in the last decades, given their wide range of possibilities for low-temperature processing compared with metals and ceramics [6]. These processing alternatives mainly focus on the depolymerization of the polymeric chains, using organic solvents, and on melting-based approaches for thin-films fabrication [8]. Solvent-assisted manufacturing methods are particularly attractive, as they are cost-effective mostly because they mainly rely on easily accessible and inexpensive equipment [3]. Moreover, this technique involves low temperatures and pressures, which is beneficial to maintain adequate and reliable mechanical responses after the addition of particulate materials when compared to thermally assisted methods, such as extrusion [9].

The use of biodegradable polymeric matrices, such as polylactides, polycaprolactones, and copolymers with natural fillers seems promising for producing novel composite biomaterials [10]. Among the most popular biopolymers, poly(lactic acid), or PLA, stands out as a linear aliphatic thermoplastic and biodegradable polymer that is relatively high strength, readily biodegradability, and easy to process [11,12,13]. However, important disadvantages include the high cost and the need for unique chemical modifications to comply with the attributes for specific applications, as is the case of developments for tissue engineering, such as regeneration of bone, cartilage, and skin, as well as the manufacture of vascular grafts [14]. Conversely, PLA has gained significant popularity as a matrix for bio-inspired composite formulation and fabrication, along with other biopolymers of the polysaccharides family, such as chitosan, alginate, and cellulose [15]. However, the mechanical properties of the obtained composites are below those of most human tissues, limiting their applicability as a medical device. This has been addressed by incorporating additional excipients into the blend, such as fillers, which include hydroxyapatite, carbon nanotubes, silica, and natural fibers [13,16,17].

In particular, natural fibers, such as manicaria, jute, rice, and peanut husk [18,19], have been used for composite materials’ development based on PLA as they exhibit improved tensile and flexural mechanical properties, lower density, and relatively lower costs than the raw polymer [20]. In consequence, the relevance of natural fibers has increased in recent years mainly due to their attractive properties, which are comparable to commonly used synthetic fibers [21]. In this regard, natural fibers from agricultural wastes have attracted special attention for their main chemical constituents (i.e., cellulose, hemicellulose, and lignin) and their wide availability worldwide, as is the case of cocoa bean shells (CBSs) [22]. In Colombia, according to the National Federation of Cocoa Growers, cocoa-production wastes were 63,416 tons during 2020 [23], which led to several environmental impacts, such as the spread of pathogenic microorganisms by their accumulation in the soil; soil pH misbalance for accumulation of heavy metals, such as nickel (Ni), cadmium (Cd), and chromium (Cr); and high levels of carbon dioxide (CO_2_) released by their main end-of-life disposal by burning [24,25,26].

From this perspective, the structural and chemical principal components of residual biomass from the agricultural industry not only represent an alternative to produce novel bio-based composite materials but is likely to serve as valorization to increase its added value [27]. In fact, recent studies on the fabrication of novel PLA-based materials by using bagasse, pineapple leaf, peanut rice, and coffee husk have demonstrated acceptable results in terms of tensile and flexural properties when compared to pristine PLA [28].

In addition to the high polysaccharide contents, the potential incorporation of natural fibers into composite materials is advantageous mainly because of their flexibility during processing, superior stiffness, low price, low density, biodegradability, high biocompatibility, and acceptable specific strength properties [29,30,31,32]. Besides these important attributes, natural fiber-based composite materials might also be a potential source for bioactive molecules, such as phenols and polyphenols, which have been reported to prevent cellular oxidative stress [33,34]. Therefore, the inclusion of natural fibers into biodegradable polymeric matrices provides a cost-effective way to formulate functional scaffolds for tissue engineering [35,36]. However, even though recent studies have demonstrated that isolated compounds from natural fibers are highly biocompatible and improve cell proliferation, their performance, when incorporated into a polymer’s matrix, is yet to be fully explored.

Accordingly, this work aims to chemically characterize raw CBS fibers by quantifying their main components and formulate, manufacture, and test PLA/CBS composites by employing spectroscopic (FTIR), thermal (TGA and DSC), and tensile mechanical characterization techniques. Then, we manufactured PLA/CBS thin-film composites by solvent casting, followed by thermal hardening, without compromising the fiber integrity. Additionally, the biocompatibility of the developed composite materials was preliminarily studied in vitro via hemocompatibility (hemolysis and platelet aggregation and activity) and cytotoxicity assays. Finally, their antioxidant activity was evaluated to investigate their potential as bioactive biomaterials.

## 2. Materials and Methods

### 2.1. Materials

Cocoa bean shells (CBSs), originally from cocoa plantations in the Andean region in Colombia, by Casa Luker S.A. (Caldas, Colombia), were collected after the harvesting, fermentation, and drying of cocoa beans. The samples were stored at room temperature in closed polypropylene re-closable zip bags. PLA 2003D TDS provided by Nature Works LLC (Minnetonka, MN, USA) was used as a polymeric matrix for the composite fabrication. Sodium chloride (NaCl, 99.9%) was obtained from PanReac AppliChem (Darmstadt, Germany). Vero cells (CCL-81) were acquired from ATCC^®^ (Manassas, VA, USA). Fetal Bovine Serum (FBS) and trypsin EDTA were obtained from Biowest (Riverside, MO, USA). Triton X-100, ethanol (99.5%), methanol (99.8%), benzene (99.8%), sulfuric acid (H_2_SO_4_, 98%), ascorbic acid (99%), Phosphate Buffer Saline (PBS), thiazolyl blue tetrazolium bromide (MTT), dimethyl sulfoxide (DMSO, 99%), Dulbecco’s modified Eagle’s medium (DMEM), glucose G8270 (99.5%), and xylose X1500 (99%) were purchased from Sigma-Aldrich (St. Louis, MO, USA). DPPH free radical was obtained from Santa Cruz Biotechnology (Santa Cruz, CA, USA).

### 2.2. CBS Sample Preparation

**Figure 1:**
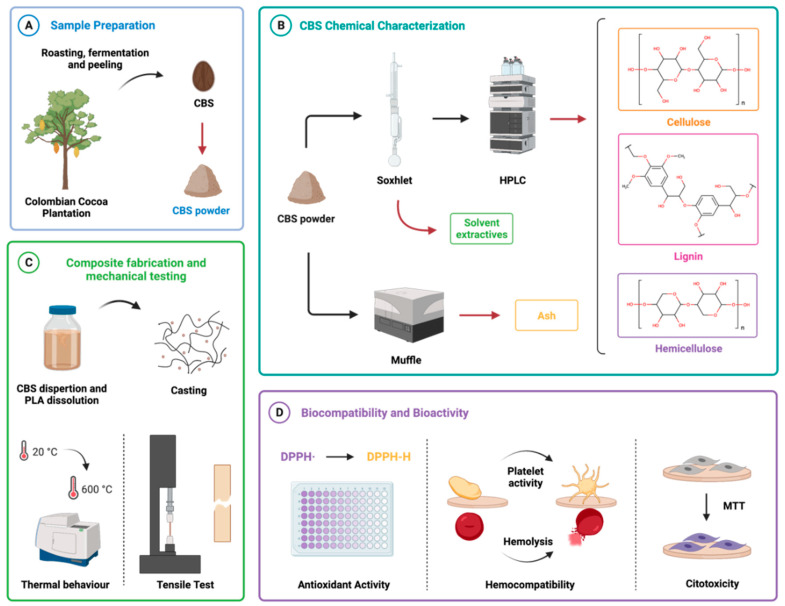
Workflow for the study of cocoa bean shells (CBSs) as filler of poly(lactic acid) (PLA) in biomaterial development. (**A**) CBSs collected after fermentation, roasting, and peeling of the cocoa bean from the Andean region of Colombia were processed by autoclaving, drying, and cryogenic grinding. (**B**) Chemical characterization by extraction and combustion was made to determine the chemical components of CBS. (**C**) Proof-of-concept for the fabrication, tensile, and thermal testing of PLA/CBS composites produced 2.5 wt.% CBS. (**D**) Preliminary biocompatibility assessment of CBS and PLA/CBS composites via DPPH radical scavenging activity, hemocompatibility (hemolysis and platelet activity), and cytotoxicity assays. Created with BioRender.com.

CBSs were autoclaved to remove microorganisms and dried at 60 °C on a forced convection oven (FD 115 Binder GmbH, Tuttlingen, Germany) overnight to remove moisture (Figure 1A). Dried CBSs were stored in a closed desiccator and subsequently pulverized with the aid of a cryogenic mill (6875 Freezer/Mill^®^, SPEX SamplePrep, Metuchen, NJ, USA). Briefly, samples of 8 g of dried CBSs were submerged in liquid nitrogen for approximately 6 min to achieve brittleness and subsequently grounded three times for 3 min, while waiting for about 1 min between grinding cycles to ensure enough brittleness. The pulverized CBSs were stored in a closed desiccator until further use.

### 2.3. CBS Chemical Characterization

CBSs’ chemical characterization was conducted to determine the main CBS components and ensure that natural polymers and solvent extractives are suitable for biomaterial reinforcement (Figure 1B).

#### 2.3.1. Extractive Content

The extractive content was quantified to estimate waxes, fats, and surface resins according to the TAPPI T204 method. A Soxhlet apparatus was used to expose the CBS powder samples to a mixture of ethanol–benzene (1:2 *v*/*v*) for 5 h after reaching its boiling point. The extractive content was then calculated from five samples, considering the dry weight ($W_{p}$) of the extract after solvent evaporation and the initial dry weight ($W_{e}$) of the powder, as shown in Equation (1).

(1) $$\%\;Extractables=\left(\frac{W_{e}-W_{p}}{W_{p}}\right)\cdot 100$$

#### 2.3.2. Lignocellulosic Content

Cellulose, lignin, and hemicellulose content of five dried extractive-free samples were determined based on the ASTM E1758 standard. Initial hydrolysis of the sample was performed by using a solution of sulfuric acid 72 wt.% (1:100 *w*/*v*) in a test tube, at 30 °C, for one hour, with mechanical stirring (250 RPM) every 15 min, and sterilized via condensation heat cycle, using an autoclave (Speedy Autoclave SX-500E, Tomy Digital Biology, Tokyo, Japan).

Lignin-soluble content was estimated by weight difference on the collected solid residues after filtering on a weighed crucible before the carbohydrate quantification. Then, cellulose and hemicellulose were quantified by measuring their structural base monomer sugars (i.e., glucose and xylose, respectively) via High-Performance Liquid Chromatography (HPLC), using an HPX-87H column (Agilent, Santa Clara, CA, USA) at 85 °C for 20 min, with 0.6 mL/min flow of deionized water and a refractive index detector. As part of the test, an independent calibration standard set was prepared with filtered analytical-grade glucose and xylose (4 mg/mL) after hydrolyzation at the previously described conditions. The percentage of each sugar on a mass basis was calculated by Equation (2).

(2) $$\%\;sugar=\left(\frac{C_{1}\cdot C_{spl}\cdot V_{f}}{C_{2}\cdot m_{1}\cdot \%T_{ext}}\right)\cdot 100\%$$

#### 2.3.3. Ash Content

CBS ash content was measured by following the ASTM E1755 standard in a Muffle (FB1310 Thermolyne^®^ BenchTop 1100 °C Muffle Furnace^®^, Thermo Scientific^®^, Waltham, MA, USA). Seven samples were heated from room temperature to 250 °C for 30 min, before increasing to 575 °C. The ash percentage was calculated based on the remaining weight and the initial dry sample.

### 2.4. Composite Preparation and Testing

#### 2.4.1. Preparation of Composites

Thin films of PLA/CBS composites containing 0 wt.% (PLA) and 2.5 wt.% (PLA/CBS 2.5 wt.%) of CBS were fabricated by solvent casting, followed by a thermal hardening to improve mechanical performance (Figure 1C). Briefly, CBS and PLA were dried in a convection oven (FD 115 Binder GmbH, Tuttlingen, Germany) overnight, at 60 °C, to eliminate the absorbed moisture. Then, CBS powder was dispersed in chloroform (1:10 *w*/*v*) at room temperature, for 24 h, to obtain a homogenous dispersion of CBS filler, and then it was sonicated for 5 min to break up agglomerates. After particle dispersion, PLA was added to the mixture and dissolved for 2 h at room temperature. The solutions were cast 24 h on glass Petri dishes for solvent evaporation in a closed glass desiccator. The resulting thin films were heated at 100 °C for 5 min on a hot plate. A total of five batches were fabricated 48 h before testing.

#### 2.4.2. Fourier-Transform Infrared Spectroscopy

CBS, neat PLA, and PLA/CBS 2.5 wt.% composite chemical groups were analyzed via Fourier-Transform Infrared Spectroscopy (FTIR) in a Bruker Alpha II FTIR Eco-ART (Bruker Optik GmbH, Ettlingen, Germany). The spectra were measured in the wavenumber range from 4000 to 600 cm^−1^ with a spectral resolution of 2 cm^−1^ and 32 scans at room temperature. Before the measurements, each sample was dried overnight at 60 °C on a forced convection oven (FD 115 Binder GmbH, Tuttlingen, Germany).

#### 2.4.3. Thermal Properties

Thermal stability and degradation profiles were determined aided by thermogravimetric analysis (TGA) according to the ASTM E1131 standard. Briefly, 4 mg of CBS, PLA, and PLA/CBS 2.5 wt.% were placed into alumina sample pans in a TA Instruments Q600 thermogravimetric analyzer^®^ (New Castle, DE, USA) and heated from 30 to 600 °C, at a rate of 10 °C/min, in a nitrogen atmosphere, by maintaining a 10 mL/min flow rate.

Further thermal analysis was undertaken by following the ASTM D3418 standard by Differential Scanning Calorimetry (DSC) analyses in a TA Instruments Q2000 (New Castle, DE, USA). Then 5 mg samples were placed into an aluminum capsule and heated at a rate of 10 °C/min, from 25 to 500 °C for CBS and from 25 to 220 °C for neat PLA and PLA/CBS 2.5 wt.% in a nitrogen atmosphere by maintaining a flow rate of 300 mL/min. Then PLA and composite specimens were cooled down to 25 °C, using the same rate and subsequently heated up to 220 °C, as described previously. From the thermograms, parameters, such as glass transition temperature (T_g_), crystallization temperature (T_c_), and melting temperature (T_m_), were determined from the second heating scan. The crystallinity was calculated by Equation (3).

(3) $$X_{c}\left(\%\right)=\frac{\Delta H_{m}^{obs}}{\Delta H_{m}^{0}}\times \frac{100}{1-w_{f}}$$

where $\Delta H_{m}^{obs}$ is the observed heat of fusion for each sample, $\Delta H_{m}^{0}$ is the heat of fusion for 100% crystalline PLA $\left(\Delta H_{m}^{0}=93\;J/g\right)$ [3], and $w_{f}$ the weight fraction of CBS fiber in the samples.

#### 2.4.4. Tensile-Strength Test

Tensile tests were carried out by following the ASTM D882 standard for the PLA and PLA/CBS 2.5 wt.% composite, using a TA.HD Plus Texture Analyzer instrument (Vienna Court, UK) equipped with a load cell of 1 kN and a crosshead speed of 8.3 mm/min. Five samples were manufactured as rectangular specimens with average dimensions of 52.2 mm × 9.4 mm × 0.06 mm for testing.

### 2.5. Biocompatibility

#### 2.5.1. MTT Cytotoxicity Assay

The cytotoxicity of CBS, PLA, and PLA/CBS 2.5 wt.% composites was carried out by quantification of the metabolic state of Vero cells (ATCC^®^ CCL-81) by measuring the conversion of MTT ((3-[4,5-dimethylthiazol-2-yl]-2,5-diphenyltetrazolium bromide) to formazan by succinate dehydrogenase in the mitochondria (Figure 1D). Briefly, a cellular density of 10.000 cells/well in DMEM medium (10% FBS, 1% PS) was deposited in a 96-well plate and incubated at 37 °C and 5% CO_2_ for 24 h. After incubation, the metabolized medium was replaced by 100 μL of fresh medium, and samples of 3 mm × 3 mm were exposed and incubated at 37 °C, in 5% CO_2_, for 24 and 72 h. Then, samples were removed, and MTT (5 mg/mL PBS) reagent was added and allowed to react for 2 h before removing the supernatant. Finally, 100 μL of DMSO was added and gently mixed to achieve the dissolution of formed formazan crystals, and the absorbance was measured at 595 nm in a microplate spectrophotometer (Thermo Scientific™, Waltham, MA, USA). DMSO and 2D cell culture were used as positive and negative controls, respectively. Finally, the cytotoxicity of five samples was calculated by subtracting the absorbance of the negative control and normalizing it by the difference of the controls (Equation (4)).

(4) $$\%Viability=\frac{Abs_{595}\left(Sample\right)-Abs_{595}\left(negative\;control\right)}{Abs_{595}\left(positive\right)-Abs_{595}\left(negative\;control\right)}\cdot 100\%$$

#### 2.5.2. Hemolysis Assay

To test potential applicability as blood-contacting material, the hemolytic behavior of the CBS, PLA, and PLA/CBS 2.5 wt.% composite was assessed as described previously by Muñoz-Camargo et al., with slight modifications, according to ISO 10993-4:2009 standard [37]. The blood samples were obtained with the approval of the Ethical Committee at the Universidad de Los Andes, minute number 928-2018, with an informed consent form signed by the subjects. Briefly, an initial sample of 4.2 × 10^6^ erythrocytes from human blood of a healthy donor was collected in an EDTA blood collection tube after plasma, and white blood cells were removed by centrifugation, at 1800 RPM, for 5 min, and washed three times with 0.9% *w/v* NaCl solution. The precipitate containing the erythrocytes was diluted 1:10 in PBS 1X, at room temperature. A sample of 100 μL of washed erythrocytes was exposed to square specimens (3 mm × 3 mm) in a 96-well microplate and incubated at 37 °C for 1 h. PBS 1X was used as the negative control, and Triton X-100 as the positive one. After incubation, the samples were removed, the microplate was centrifuged for 5 min at 1800 RPM, and the absorbance of each supernatant was measured at 450 nm in a microplate spectrophotometer (Thermo Scientific™, Waltham, MA, USA). The hemolysis percentage was calculated by subtracting the absorbance of the negative control from the tested sample and normalizing it by the difference of the controls, as shown in Equation (5). A total of five samples of each treatment were exposed and measured.

(5) $$\%Hemolysis=\frac{Abs_{450}\left(Sample\right)-Abs_{450}\left(negative\;control\right)}{Abs_{450}\left(positive\right)-Abs_{450}\left(negative\;control\right)}\cdot 100\%$$

#### 2.5.3. Platelet Aggregation Assay

Platelet aggregation induced the by CBS, PLA, and PLA/CBS 2.5 wt.% composites was tested by the precipitation of platelets isolated from a human blood sample of a healthy donor collected in a vacutainer tube with 3.2% sodium citrate [12]. The sample was centrifugated at 1000 RPM for 15 min to obtain platelet-rich plasma (PRP). A sample of 100 μL of PRP was placed on a microplate and allowed to have contact for 3 min with square samples (3 mm × 3 mm). The positive control was 70 μL of PRP and 30 μL of epinephrine, while the negative was 70 μL of PRP and PBS (1X). Finally, 50 μL of each supernatant was transferred to a 96-well microplate, and the absorbance was read at 620 nm in a microplate spectrophotometer. A total of five samples were tested.

#### 2.5.4. Platelet Activity

Quantification of platelets activity was determined by measuring the enzymatic activity of the lactate dehydrogenase (LDH) released by adherent platelets after exposure to composites. PRP containing 6.5 × 10^6^ platelets was isolated from a healthy human donor in a vacutainer tube with sodium citrate by centrifugation, at 1000 RPM, for 15 min. Square samples (3 mm × 3 mm) were exposed to 100 μL of PRP in a 96-well microplate, for 1 h, at 37 °C, and then washed with PBS 1X. Washed samples were transferred to a new microplate, and adherent platelets were lysed with Triton X-100 by allowing exposure for 15 min. Then, supernatants were extracted and exposed to LDH Detection Kit (Roche, Basel, Switzerland) and allowed to react for 5 min at room temperature, in the dark. PBS 1X was used as a negative control, and Triton X-100 as a positive control. After incubation, absorbance was read at 620 nm in a microplate spectrophotometer. In addition, for platelet quantification, a calibration curve was made, replacing the treatments with serial dilutions (1:2 *v/v*) of PRP (6.5 × 10^6^ to 4.1 × 10^5^ platelets) and subsequently measuring its absorbance to fit a linear regression, using the least-squares approach ($R^{2}=0.99$) and linear dependency of $y=1\times 10^{-7}x+0.37$. A total of five samples were exposed. Absorbance (y) was included in the regression model equation to obtain the platelet number present in each sample, and then it was normalized to the samples’ area (9 mm^2^).

### 2.6. Antioxidant Activity

CBS’s antioxidant activity was determined by measuring DPPH (2,2-diphenyl-1-picryl-hydrazyl-hydrate) radical scavenging activity of methanol extracts, as described previously [38,39]. Briefly, CBS and autoclave-sterilized CBS (S-CBS) extracts were prepared by homogenizing dried samples in methanol (1:1 *w*/*v*), at 12,000 RPM, for 1 min, in an ice-water bath. The mixture was subsequently filtered through filter paper (Whatman No. 1), under reduced pressure, to isolate the liquid phase and then concentrated by using a rotary evaporator at 37 °C and 100 RPM to remove excess solvent. Then, 100 μL samples of serial dilutions of a concentrated stock of methanol extracts (1000 to 7.81 µg/mL) were added to 0.2 mM DPPH methanolic solution in a 96-well microplate and incubated for 30 min, at 37 °C, in the dark. Ascorbic acid (AA) was used as the standard, at different concentrations (1000 to 7.81 µg/mL). The absorbance was recorded at 517 nm, using a microplate spectrophotometer (Thermo Scientific™, Waltham, MA, USA), and DPPH radical scavenging activity (%) was calculated according to Equation (6) to assess the antiradical potency (EC50), which is defined as the concentration of substrate that causes 50% reduction of DPPH color. Finally, the antioxidant radical power (ARP) was calculated as the inverse of EC50 value. A total of three samples were tested.

(6) $$\%\;\mathrm{DPPH}=\frac{A_{517}\left(\mathrm{DPPH}\right)-A_{517}\left(Sample\right)}{A_{517}\left(\mathrm{DPPH}\right)}\cdot 100\%$$

In addition, the PLA and PLA/CBS 2.5 wt.% composite’s antioxidant activity was evaluated by exposing five square samples (3 mm × 3 mm) to 300 µL of methanol for 8, 12, 24, and 48 h, at 37 °C, under continuous agitation. After incubation, 100 µL of each medium was transferred to a new 96-well microplate and exposed to 0.2 mM methanolic DPPH solution, as described previously.

### 2.7. Statistical Analysis

Properties and compositions found throughout this study were expressed as means and standard deviations. In addition, the one-way ANOVA test was implemented with a 95% confidence level (*p*-value < 0.05) to *establish* the statistical significance of the experimental results. Multiple comparisons were made for samples against neat PLA, using statistical hypothesis testing by a Tukey test implemented in GraphPad Prism^®^ 9 software (San Diego, CA, USA). In all cases, ANOVA assumptions were conducted (i.e., tests for normality, independence of observations, and homoscedasticity) before the test.

## 3. Results and Discussion

### 3.1. CBS Characterization

#### Chemical Composition

CBSs’ main components were characterized by measurements of the lignocellulosic fiber content, given its vegetable fiber character, and the results are shown in Table 1. In general, CBS was found to be mainly composed of cellulose (42.23 ± 1.93 wt.%), lignin (22.68 ± 1.17 wt.%), hemicellulose (14.73 ± 1.57), and solvent-extractives (14.42 ± 1.94), leading to its classification as grass and reed fiber [40,41]. A mass balance indicates that no other chemical components are present in CBS. Therefore, CBS’s composition makes it suitable as a composites filler, since mechanical properties mainly depend on the cellulose content [42,43]. Nevertheless, lignin content may affect fiber-matrix compatibility, given the increase of hydrophilicity, which could be avoided by a fiber pretreatment [44].

Table 1 presents the comparison of chemical components reported for different lignocellulosic fibers, including CBS. On average, CBS has 7.69% more cellulose than coffee, wheat, and rice husks and content similar to that of peanut husk. On average, lignin, hemicellulose, and ash CBS contents approached those of similar vegetable fibers (Table 1). However, CBS solvent-extractives (i.e., fats, resins, polyphenols, vitamins, and proteins) are higher than those of peanut and rice husks [47]. This likely indicates that CBS contains higher amounts of low-molecular-weight carbohydrates, such as flavonoid polyphenols, with demonstrated antioxidant, anticancer, and anti-inflammatory activities, as described by Lee et al. [36] and Mira et al. [50].

The chemical composition characterization reported here suggests that CBSs represent not only a source for the extraction of their main components, but also an alternative to improve the biochemical surface properties of inert polymers [45,51]. A polysaccharide-based structure with more than 50% of fiber contents (i.e., cellulose and hemicellulose) likely indicates that, upon CBS incorporation into biodegradable polymers, biocompatibility and biodegradability might increase significantly. This is without sacrificing mechanical properties and the inherent ability to mimic the extracellular matrix (ECM) [52]. This supports the notion of CBS’s potential applicability as bioactive fiber-reinforcement in polymer matrixes for three-dimensional (3D) networks for applications in tissue engineering, primarily due to the cellulose and solvent-extractive contents.

### 3.2. Spectroscopic Characterization

**Figure 2:**
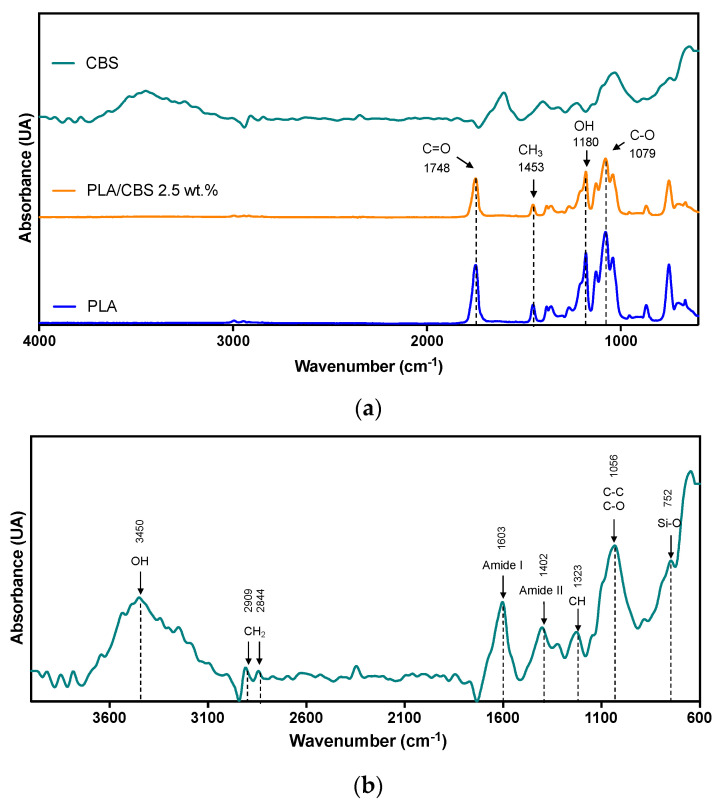
(**a**) FTIR spectra of CBS fiber, neat PLA, and PLA/CBS 2.5 wt.% measured in frequency a range between 4000 and 600 cm^−1^. Arrows highlight representative peaks for amorphous PLA in 1748, 1453, 1180, and 1079 cm^−1^ present in all samples. (**b**) Magnification of CBS FTIR spectra from 4000 to 600 cm^−1^, with arrows at peaks attributed to main constituents of the fiber (cellulose, hemicellulose, and lignin).

The FTIR analysis of the CBS powder, neat PLA, and PLA/CBS composite confirmed the presence of the main components of the CBS, corresponding to cellulose, hemicellulose, and lignin (Figure 2b). The main peak located at 3450 cm^−1^ can be attributed to the stretching vibration of the hydroxyl group (OH) in polysaccharides, while the absorbed moisture was observed in the range between 3000 and 3800 cm^−1^. Moreover, the bands at 2909 and 2844 cm^−1^ correspond to asymmetric and symmetric stretching vibrations from lipids [13,53,54]. Moreover, peaks at 1603 and 1402 cm^−1^ were attributed to amides I and II of proteins present in the materials [55] and bending vibrations of methylene groups of cellulose [21]. Furthermore, the band identified at 1323 cm^−1^ belongs to C-H stretching vibration of lignin, and the band at 1056 cm^−1^ was associated with C-C and C-O stretching of polysaccharides and lignin [56]. In addition, according to characterization studies of cellulosic fibers by spectroscopy, absorption bands between 900 and 700 cm^−1^ are attributed to C-H bending vibrations of lignin [57]. In general, the CBS spectra show a good agreement with previous reports of the same agricultural residue [22,58].

FTIR analysis of the PLA and PLA/CBS 2.5 wt.% composite (Figure 2a) was carried out to investigate chemical interactions between the matrix and the filler and demonstrated that only blending occurred, as evidenced by the similarity of the collected spectra. Moreover, the results strongly indicate that CBS incorporation led to no significant changes in the molecular structure of PLA [25]. Similar results are found in the literature for PLA/natural fiber composites [55]. Further, the PLA/CBS 2.5 wt.% composite and neat PLA peaks at 1748 and 1453 cm^−1^ can be attributed to the C=O stretching vibration of ester and -CH_3_ present in amorphous PLA, respectively [26]. Typical neat PLA peaks at 1180 and 1079 cm^−1^ were assigned to -OH bending vibrations and C-O stretching vibrations. Small peaks in the range of 1250–950 cm^−1^ in all samples were related to C-O-C functional group [25].

### 3.3. Thermal Properties

**Figure 3:**
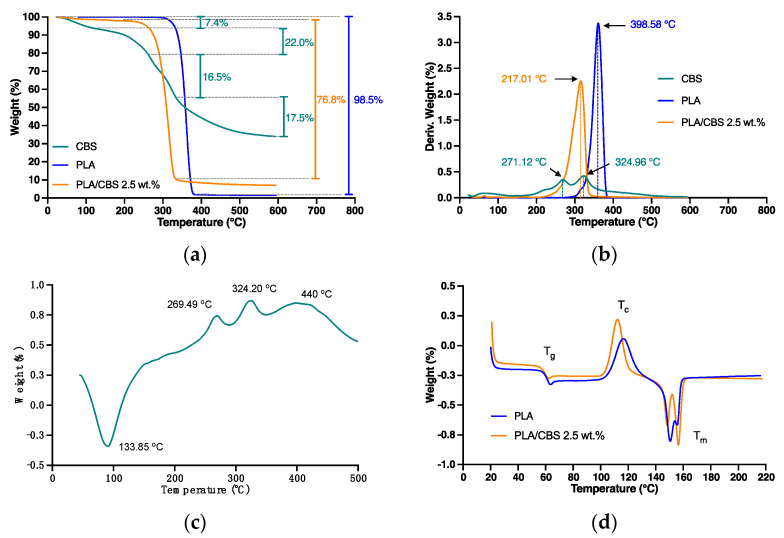
Thermal characterization of CBS, PLA, and PLA/CBS composites. (**a**) TGA analyses of CBS, PLA, and PLA/CBS 2.5 wt.% composite. Weight-loss percentage is presented in each case. (**b**) DTGA curves of CBS, PLA, and PLA/CBS 2.5 wt.%. The main degradation peaks of CBS and PLA are indicated in the thermogram. (**c**) DSC curve of CBS. Major endothermic and exothermic peaks are indicated in the thermogram. (**d**) DSC analyses of PLA/CBS 2.5 wt.% composite. Glass transition (T_g_), crystallization (T_c_), fusion (T_m_), and temperatures are indicated in the thermogram.

The thermal analyses (TGA) that were performed to determine the differences in thermal stability between CBS fiber, PLA, and CBS/PLA 2.5 wt.% composite in a nitrogen atmosphere are presented in Figure 3. In addition, DTGA curves were calculated from the TGA as the rate of material mass change with respect to temperature to facilitate identifying the degradation thermographs peaks. Thermal degradation (Figure 3a,b) of neat PLA occurred in a single mass-loss step, starting at 330 °C, obtained by the extrapolation of the slope of the DTGA curve (Figure 3b) by connecting the maximum peak to the zero level on the *x*-axis [59].

In general, CBS fiber displayed three main weight loss steps (Figure 3a,b). The first weight loss of 5% between 50 and 100 °C can be attributed to moisture evaporation. The second decomposition (25%) took place between 220 and 290 °C, due to hemicellulose degradation [60], followed by the third decomposition (37%), which was between 290 and 530 °C and attributed to the degradation of cellulose and lignin [38]. In addition, a remaining residual char of 34% in CBS was observed as a product of the pyrolysis process. Moreover, the overall TGA profile shows typical thermal behavior for lignocellulosic fibers and similar weight losses and residual mass observed previously for Italian CBS [61].

The TGA profile of the PLA/CBS composite exhibits lower temperature stability upon CBS incorporation as evidenced by a decrease of about 58 °C in the onset temperature of decomposition from 330 to 272 °C, in comparison with PLA. This thermal stability reduction after including the natural fiber to form the polymeric composites is most likely related to the maximum allowable temperature for processing the raw fiber, which is usually lower than the pristine polymer [60]. This behavior indicates that an increase in natural fiber weight ratio will cause a decrease in the thermal stability of the composite, approaching those of the first degradation found in the fiber’s TGA profile [32].

This maximum allowable temperature largely defines further processability requirements of the composite to avoid possible degradation. In addition, a residual solid mass percentage detected at the final temperature (600 °C) was around 2.5%, which corroborates the CBS weight ratio in the composite. Finally, the thermal behavior of the PLA/CBS composite shows results similar to those of PLA composites with natural fiber fillers [60,62,63].

According to thermal data shown in Table 2 and *the* DSC curve of CBS in Figure 3c, major endothermic and exothermic peaks are found to be closely related to the main fiber components: hemicellulose, cellulose, and lignin. From 45 to 190 °C, an endothermic peak occurs with a gradual decrease of heat flow given the energy necessary for water evaporation commonly retained in natural fibers [64]. In addition, two exothermic degradation peaks are observed where hemicellulose and cellulose decompose with maximum values at 269 and 324 °C, respectively. This is followed by the formation of flammable, volatile products, as has been described in previous studies with isolated cellulose and hemicellulose specimens and their splitting hydroxyl groups [65,66]. Furthermore, the weak exothermic final peak with a starting temperature around 356 °C and a maximum value at 440 °C is attributed to lignin degradation [65]. The results obtained by DSC analysis of CBS fiber and its chemical components were found to be comparable to those achieved by previous authors with sisal, coconut, and fique fibers [67,68].

Figure 3d shows the DSC thermograms of PLA and PLA/CBS 2.5 wt.% composite, and the corresponding thermal parameters are presented in Table 3. The T_g_ values of PLA and PLA/CBS 2.5 wt.% slightly decrease with the addition of CBS from 63.63 to 62.51 °C, and this decrease can be attributed to the reduction of the interaction between PLA polymer chains and CBS, resulting in a plasticization effect to induce the movement of polymer chains. The crystallization peak tends to decrease with fiber incorporation, due to the nucleating effect of CBS, which accelerates the crystallization process of PLA [69]. Thus, the addition of CBS improved the crystallinity of PLA, due to the same CBS nucleating effect, which is in line with the work by Wei et al. that showed superior crystallinity of natural fiber composites compared with pristine PLA mainly due to the ability of some fiber components to induce the formation of new crystals [11]. Additionally, the two melting peaks of the evaluated materials provide evidence of different PLA crystals, which can be related explicitly to α-monocrystals formed during PLA heating [65,70]. The melting temperature (Tm) of the PLA/CBS 2.5 wt.% experienced a slight decrease of about 2% compared to neat PLA. This behavior can also be explained by the fiber’s nucleating effect that tends to form incomplete crystalline structures [69].

In summary, the thermal behavior of PLA/CBS composite holds much promise regarding the possible processing of the fabricated composite by versatile heat-processing methods for rapid prototyping, including extrusion, compression molding, and even 3D printing. This considering that CBS incorporation leads to no observable changes in the thermal stability of the pristine polymer.

### 3.4. Composite Mechanical Properties

**Figure 4:**
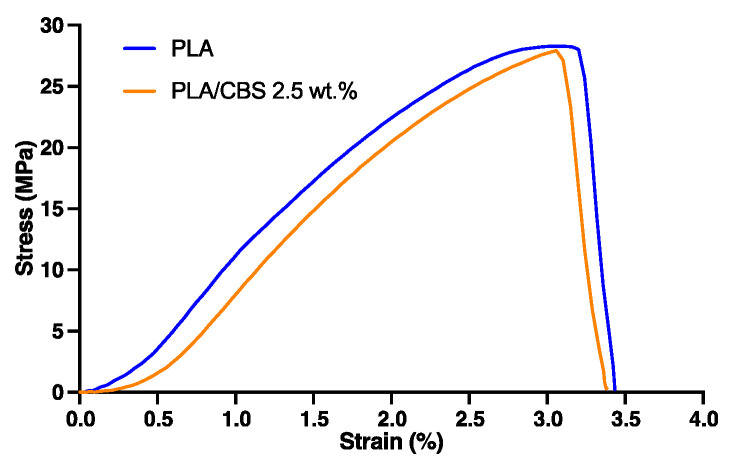
Representative stress–strain tensile curves of PLA and PLA/CBS 2.5 wt.% composite.

Tensile properties of PLA and PLA/CBS 2.5 wt.% composite are summarized in Table 4. A representative stress–strain curve is shown in Figure 4 with common elastic and plastic regions. Tensile strength and percent elongation of PLA was 30.50 MPa and 3.46%, respectively. The results indicate that incorporation of CBS fiber at the tested weight ratio (i.e., 2.5 wt.%) shows no significant impact on the tensile performance of PLA. In addition, the low dispersion of tensile data, as evidenced by their low standard deviation, indicates a homogeneous dispersion of particles. A similar behavior for the young’s modulus has been reported previously for PLA/oat-husk composites [10]. However, a decrease in tensile properties is expected for a higher CBS weight ratio, since intercalation of filler within the polymer’s molecular chains might alter intermolecular forces, therefore causing a loose chain entanglement [13].

The stiffness of PLA slightly increased with the addition of CBS, as evidenced by the change in the tensile modulus, which increased by 2.45% from 3.33 GPa for the neat PLA to 3.45 GPa for PLA/CBS 2.5 wt.% composite. This stiff behavior seems to be corroborated by the no significant changes in the elongation at the breaking point at the tested CBS weight ratio (*p* < 0.05). According to Murariu et al. [71], this behavior is explained by the reduced polymer chain mobility due to the limited polymer-matrix and fiber interactions, evidenced by an increase in the Young’s Modulus and the decrease in tensile strength [72]. Thus, the obtained tensile properties can present an appropriate alternative to introducing an enhanced performance of polymer-based materials with tunable mechanical properties (mainly dependent on the filler weight ratio) for mimicking tissue microenvironments more accurately. PLA/CBS composites behavior seems to exhibit mechanical properties that resemble those of human trabecular bone [73], which are also comparable with those of commonly used PLA composite biomaterials, such as PLA/Chitosan [74] and PLA/Hydroxyapatite [75].

### 3.5. Biocompatibility

Biocompatibility was tested to assure that the developed materials pose no potential hazards and are safe for human use. This is critical for their eventual translation into in vivo preclinical and clinical applications [76,77,78]. Hence, in vitro cytotoxicity, hemolytic activity, and platelet aggregation tendency of raw materials (PLA and CBS) and the PLA/CBS 2.5 wt.% composite were evaluated.

**Figure 5:**
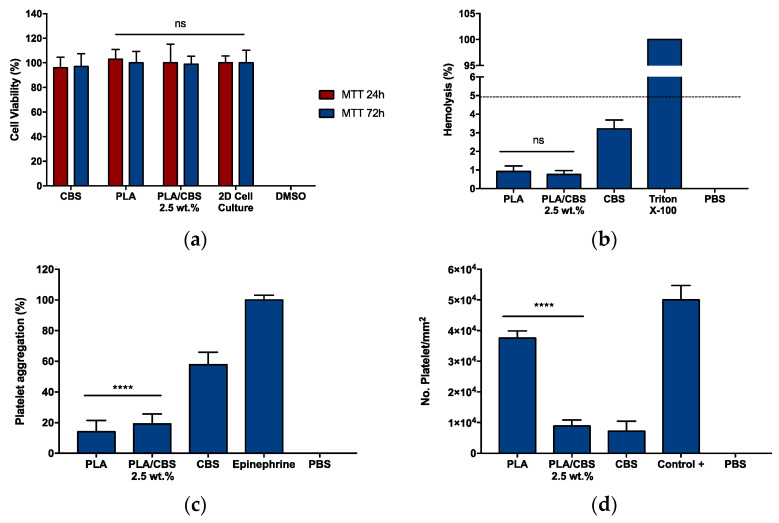
Biocompatibility assays of CBS, neat PLA, and PLA/CBS 2.5 wt.% composites. (**a**) MTT cytotoxicity in Vero cell line after 24 and 72 h. Cell viability remains constant above 94%. (**b**) Hemolytic behavior after an incubation time of 1 h. The hemolytic effect was below 5%, complying with the ISO 10993-4 standard requirement in all cases. Triton X-100 and PBS were used as positive and negative controls, respectively. (**c**) Platelet aggregation assay. Epinephrine and PBS were used as positive and negative controls, respectively. (**d**) Platelet activity was measured after 1 h of exposure. Thrombin and PBS were used as positive and negative controls, respectively. ANOVA one-way with a confidence level of 95% results are shown, with “****” for *p*-values less than 0.05 and “ns” for a non-statistically significant difference.

Following MTT cytotoxicity assay (Figure 5a,b), in all cases, Vero cells were metabolically active with cell viability levels above 94% after 24 and 72 h of exposure to the treatments. The negligible impact on cell viability of our composites agrees well with cytotoxicity assays reported previously for cocoa- and parsley-derived fibroin [35,79] and are superior to those obtained for bagasse [15]. This verifies that the incorporation of CBS into the PLA matrix was not detrimental for the cells, and it could be potentially employed as a bioactive filler in smart biomaterial development. Accordingly, the low cytotoxicity of our composites can be explained by the main chemical composition of the CBS that, in general, is similar to the proteins and polysaccharides present in the extracellular matrix (ECM) from different human tissues [79].

To test the potential applicability of our composite as blood-contacting materials, erythrocytes lysis (Figure 5c) and platelet aggregation (Figure 5d) were measured. The percentage of erythrocytes lysis in the presence of PLA was close to previous studies where PLA was used as a control for biomaterial development and functionalization [76,80,81]. The hemolytic performance of CBS is comparable to similar studies with rice-rusk- and plant-derived cellulose and lignin [82,83]. In comparison with PLA, the hemolysis induced by the composite appears to increase, approaching the hemolytic behavior of CBS with non-significant changes when a *p*-value of 0.05 is used. This might be explained by the increase of polar hydroxyl groups related to the lignocellulosic material and the presence of surface irregularities [84,85]. However, all tested samples can be considered non-hemolytic, since the hemolysis percentages are below the maximum permissible limit (5%) allowed by the ISO 10993-4 standard [85].

Platelet aggregation percentages were above 50% for CBS (Figure 5c). The PLA/CBS 2.5 wt.% composite exhibited an incremental aggregation tendency from PLA to CBS of 6.6%. These results can be attributed to the hydrophilicity and non-electrostatic repulsion induced by CBS, leading to platelet adhesion [86]. However, platelet activity after 1 h of exposure and before adherent platelets lysis (Figure 5d) demonstrated detachment over time, indicating that the reversible aggregation is likely to occur, therefore limiting platelet activation [87]. Moreover, platelet detachment over time seems to be decreased when CBS is included, possibly due to the presence of polar hydroxyl groups on the lignocellulosic surface and the antioxidant activity of CBS that avoids platelet activation via oxygen reactive species (ROS) stabilization [88], as is discussed below. Overall, PLA/CBS composite materials seem promising for decreasing platelet aggregation over time, which is attractive to formulate blood-contacting materials [89,90,91]. Finally, the obtained results confirm the high biocompatibility of the PLA/CBS composite in terms of MTT cytotoxicity and hemocompatibility and pave the way for the use of CBS as biocompatible fillers for sustainable and bioactive biomaterial formulation with application in the biomedical field.

### 3.6. Antioxidant Activity

**Figure 6:**
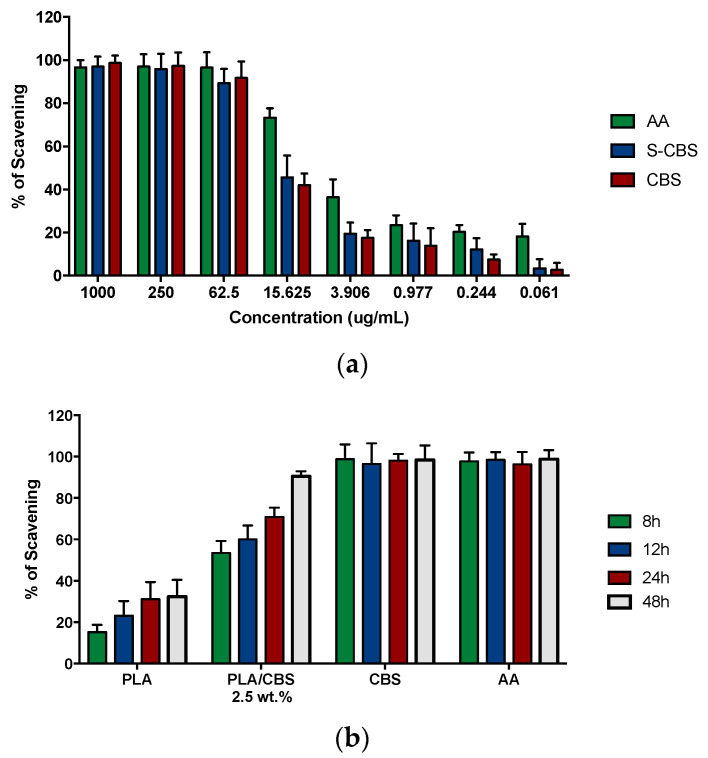
(**a**) DPPH radical scavenging activity (%) of CBS and sterilized CBS (CBS-S) at different concentrations. Ascorbic acid (AA) was used as the standard for high antioxidant activity. (**b**) DPPH radical scavenging activity (%) of PLA/CBS 2.5 wt.% composite. After 24 h, the RSA of composites seems to be effective in obtaining EC_50_. AA and CBS were included as a control for RSA over time.

DPPH free radical scavenging capacity by the antioxidant activity of CBS and sterilized CBS (S-CBS) by autoclave are shown in Figure 6. The obtained EC50 of methanolic extracts of untreated CBS (4.14 ± 0.028) and sterilized CBS (5.08 ± 0.046) show no statistically significant differences (*p*-value < 5%), demonstrating that autoclave sterilization had no impact on the antioxidant activity. Moreover, CBS and S-CBS antiradical capacity shows strong DPPH scavenging activity (99.02 ± 0.023% and 98.77 ± 0.034%, respectively) [92]. These results are attributed mainly to phytochemicals in the extracts, such as polyphenols, carotenoids, flavonoids, tannis, and procyanidin B1 and B2 [55,93].

A comparison of the DPHH radical scavenging activity of the samples with the internal standard antioxidant (ascorbic acid) in terms of EC50 and ARP values is presented in Table 5. Since it is reported that antioxidant activity is more potent for high ARP values and low EC_50_ ones, CBS exhibits stronger action than ascorbic acid (vitamin C) [94,95]. Therefore, besides high biocompatibility, CBS is attractive for biomaterial development, considering the possibility of regulating free-radical-caused cell damage and immunomodulatory responses mediated by reactive oxygen species (ROS) in trauma locations [96]. This is the case for bone-resorption processes during bone repair [18]. In addition, the antioxidant properties of CBS position this agricultural waste as a potential source of bioactive compounds.

The radical scavenging activity (RSA) of methanolic extracts of PLA and PLA/CBS 2.5 wt.% at four time points (8, 12, 24, and 48 h) after extraction is shown in Figure 6b. PLA shows low RSA against DPPH, which can be attributed to the monomer decomposition reaction that interferes with the amine radical group of the DPPH reagent [97]. In contrast, the inclusion of CBSs into the polymer matrix led to increased antioxidant properties. In general, the RSA of composites increases as a function of the CBS content, and the EC50 of DPPH for raw CBSs is reached after 48 h of exposure with an average incremental value between consecutive time points of 21.3%. These results confirm that, even if particles remain trapped into a polymeric matrix, their antioxidant properties remain effective, as is consistent with studies for natural fiber composites reported previously [98]. In this scenario, the prepared composites could be further exploited as agents for ROS, platelet activation, and immune response control [87].

## 4. Conclusions

The use of natural fiber-based composite materials has emerged as a promising alternative for next-generation biomaterial fabrication. In fact, the incorporation of fibers into biodegradable polymers provides structural reinforcement and improves their surface bioactivity. Here, we introduced the fabrication of a novel biodegradable thin-film composite based on PLA and Colombian CBS. To our knowledge, the present study provides, for the first time, a route for the use of CBS as reinforcement and to improve the bioactivity of synthetic polymeric materials in biomedical research.

As expected by their vegetal origin, the main components of CBS are cellulose, lignin, and hemicellulose. The tensile test included to analyze mechanical properties of the composite preliminarily confirmed that CBS is suitable as filler for the PLA matrix at a low content (2.5 wt.%). However, further testing increasing the CBS weight ratio needs to be conducted for a final interrogation of mechanical performance. TGA and DSC thermograms revealed the possibility of implementing several processing alternatives, including compression molding, extrusion, and even 3D printing. These versatile technologies provide various avenues for novel 3D scaffold fabrication with complex topologies and functionalities, as they are increasingly demanded for most recent tissue-engineering applications.

Bioactivity assessed by measuring the antioxidant capacity of CBS and the corresponding composite with PLA demonstrated a marked radical-scavenging activity. We hypothesized that this might be related to the presence of polyphenols, flavonols, and tannis, as they have been reported previously in cocoa and CBS. Finally, cytotoxicity via MTT showed a viability reduction of only 10%, and a hemolysis assay indicated only 2% of erythrocyte lysis. A platelet activity assay showed that CBS incorporation is likely to induce platelet detachment over time.

In summary, the formulated and manufactured composites hold a significant promise for eco-friendly, low-cost, structurally robust, and bioactive alternative biomaterial development. This could be further exploited to overcome the lack of bioactivity and low mechanical performance of traditional polymeric materials employed as regenerative biomaterials for bone and cartilage tissues.

## Figures and Tables

**Table 1:** Chemical composition of different fibers.

Shell	Chemical Composition (% *w*/*w*)					Reference
	Cellulose	Hemicellulose	Lignin	Ash Content	Solvent Extractives	
Cocoa Bean	42.23 ± 1.93	14.73 ± 1.57	22.68 ± 1.17	9.11 ± 0.24	14.42 ± 1.94	This study
Rice	31.12	22.48	22.34	13.87	2.33	[45]
Coffee	36.50	19.00	16.50	7.75	-	[46,47]
Wheat	36.00	18.00	16.00	6.50	-	[48]
Peanut	44.80	5.60	33.24	4.80	5.50	[49]
Average	37.10	16.27	22.02	8.98	3.91	-
Deviation	4.91	6.38	6.94	3.68	1.58	-

**Table 2:** Thermal data from DSC thermal curve of CBS fiber and its constituents.

Component	Initiation Peak Temperature (°C)	Peak Temperature (°C)
Moisture ^a^	45	134
Hemicellulose	242	271
Cellulose	291	328
Lignin	356	440

^a^ Endothermic peak.

**Table 3:** DSC data of PLA and PLA/CBS composites.

Component	T_g_ (°C)	T_c_ (°C)	T_m_ (°C, 1st)	T_m_ (°C, 2nd)	ΔH_c_ (J/g)	ΔH_m_ (J/g)	X_c_ (%)
PLA	63.63	116.58	150.60	155.58	28.23	24.10	25.91
PLA/CBS 2.5 wt.%	62.51	112.22	148.42	156.48	28.91	28.31	32.04

**Table 4:** Mechanical properties of neat PLA and PLA/CBS 2.5 wt.% composite.

Mechanical Properties	PLA	PLA/CBS 2.5 wt.%
Tensile strength (MPa)	30.50 ± 1.52	27.7 ± 1.91
Tensile elongation (%)	3.46 ± 0.17	3.34 ± 0.36
Toughness (MJ/m^3^)	25.21 ± 0.5	26.02 ± 0.23
Young’s modulus (GPa)	3.33 ± 0.10	3.45 ± 0.08

**Table 5:** Comparison of DPPH radical scavenging antioxidant activity.

Compound	EC_50_ (μg/mL)	ARP
CBS	18.56 ± 0.028	0.053
S-CBS	17.08 ± 0.046	0.058
Ascorbic Acid	10.72 ± 0.069	0.093

## Data Availability

The data presented in this study are available on request from the corresponding author.
